# Ultraconserved Non-coding DNA Within Diptera and Hymenoptera

**DOI:** 10.1534/g3.120.401502

**Published:** 2020-06-29

**Authors:** Thomas Brody, Amarendra Yavatkar, Alexander Kuzin, Ward F. Odenwald

**Affiliations:** * Neural Cell-Fate Determinants Section,; † Division of Intramural Research, Information Technology Program, NINDS, NIH, Bethesda, Maryland

**Keywords:** Ultraconserved non-coding sequences, EvoPrinter, Enhancers

## Abstract

This study has taken advantage of the availability of the assembled genomic sequence of flies, mosquitos, ants and bees to explore the presence of ultraconserved sequence elements in these phylogenetic groups. We compared non-coding sequences found within and flanking *Drosophila* developmental genes to homologous sequences in *Ceratitis capitata* and *Musca domestica*. Many of the conserved sequence blocks (CSBs) that constitute *Drosophila cis*-regulatory DNA, recognized by *EvoPrinter* alignment protocols, are also conserved in *Ceratitis* and *Musca*. Also conserved is the position but not necessarily the orientation of many of these ultraconserved CSBs (uCSBs) with respect to flanking genes. Using the mosquito *EvoPrint* algorithm, we have also identified uCSBs shared among distantly related mosquito species. Side by side comparison of bee and ant *EvoPrints* of selected developmental genes identify uCSBs shared between these two Hymenoptera, as well as less conserved CSBs in either one or the other taxon but not in both. Analysis of uCSBs in these dipterans and Hymenoptera will lead to a greater understanding of their evolutionary origin and function of their conserved non-coding sequences and aid in discovery of core elements of enhancers.

This study applies the phylogenetic footprinting program *EvoPrinter* to detection of ultraconserved non-coding sequence elements in Diptera, including flies and mosquitos, and Hymenoptera, including ants and bees. *EvoPrinter* outputs an interspecies comparison as a single sequence in terms of the input reference sequence. Ultraconserved sequences flanking known developmental genes were detected in *Ceratitis* and *Musca* when compared with *Drosophila* species, in *Aedes* and *Culex* when compared with *Anopheles*, and between ants and bees. Our methods are useful in detecting and understanding the core evolutionarily hardened sequences required for gene regulation.

Phylogenetic footprinting of *Drosophila* genomic DNA has revealed that *cis*-regulatory enhancers can be distinguished from other essential gene regions based on their characteristic pattern of conserved sequences (Hardison 2000; Bergman *et al.* 2002; Odenwald *et al.* 2005; Pennacchio *et al.* 2006; Brody *et al.* 2007; Kuzin *et al.* 2009; Kuzin *et al.* 2012). Cross-species alignments have also identified conserved non-coding sequence elements associated with vertebrate developmental genes (Thomas *et al.* 2003; Bejerano *et al.* 2005), and sequences that are conserved among ancient and modern vertebrates (*e.g.*, the sea lamprey and mammals). Elements conserved between disparate taxa are considered to be ‘ultraconserved elements’ (Irvine *et al.* 2002; Visel *et al.*, 2008; McEwen *et al.* 2009; Maeso *et al.* 2012). Many of these sequences act as *cis*-regulators of transcription (Pennacchio *et al.* 2006; Irvine *et al.* 2002; Visel *et al.* 2008; Visel *et al.* 2013; Dickel *et al.*, 2018). Previous studies have identified ultra-conserved elements in dipterans, *Drosophila* species and sepsids and mosquitos (Glazov *et al.* 2005; Hare *et al.* 2008; Sieglaff *et al.* 2009 Engstrom *et al.*, 2007; Tan *et al.*, 2019). Comparison of consensus transcription factor binding sites in the spider *Cupiennius salei* and the beetle *Tribolium castaneum* have been shown to be functional in transgenic *Drosophila* (Ayyar *et al.* 2010).

In this study, we describe sequence conservation of non-coding sequences within and flanking developmentally important genes in the medfly *Ceratitis capitata*, the house fly *Musca domestica* and *Drosophila* genomic sequences (Table 1). The house fly and Medfly have each diverged from *Drosophila* for ∼100 and ∼120 My respectively (Beverley and Wilson, 1984). Our analysis reveals that, in many cases, CSBs that are highly conserved in *Drosophila* species, as detected using the *Drosophila EvoPrinter* algorithm, are also conserved in *Ceratitis* and *Musca*. Additionally, the linear order of these ultraconserved CSBs (uCSBs) with respect to flanking structural genes is also maintained. However, a subset of the uCSBs exhibits inverted orientation relative to the *Drosophila* sequence, suggesting that while enhancer location is conserved, their orientation relative to flanking genes is not.

**Table 1:** Genomic regions analyzed for presence of uCSBs

Insect Order	Species	Gene	Genomic Location	Conserved Sequence Blocks	Figure
Diptera	*Drosophila melanogaster (Dm)*, *Musca domestica (Md)*, *Ceratitis capitata (Cc)*	*ventral veins lacking/prat**^a^* intragenic	*Drosophila melanogaster* chr3L: 6,821,518-6,823,267 spanning 1,749 bp	*Musca domestica* - 3 uCSBs*^b^*	Fig 1
				*Ceratitis capitata* - 4 uCSBs	
Diptera	*Dm*, *Md*, *Cc*	*ventral veins lacking/prat2**^a^* intragenic	*Drosophila melanogaster* chr3L: 6,816,217-6,837,478 intragenic spanning 21,261 bp	*Musca domestica* - 8 uCSBs	Fig S1
				*Ceratitis capitata - 8* uCSBs	
Diptera	*Dm*, *Md*, *Cc*	*homothorax*	*Drosophila melanogaster* chr3R:10,558,002-10,613,102 upstream & intronic spanning 55,100 bp	*Musca domestica* - 16 uCSB6	Not shown
				*Ceratitis capitata* - 17 uCSBs	
Diptera	*Dm*, *Md*, *Cc*	*homothorax*	*Drosophila melanogaster* chr3R:10,612,883-10,613,947 upstream & intronic spanning 1,064 bp	*Musca domestica* - 3 uCSBs	Fig S2
				*Ceratitis capitata* - 3 uCSBs	
Diptera	*DM*, *Md*, *Cc*	*goosecoid*	*Drosophila melanogaster* chr2L:583,290-599,309 upstream & intronic spanning 16,029 bp	*Musca domestica* - 4 uCSBs	Not shown
				*Ceratitis capitata* - 6 uCSBs	
Diptera	*Drn*, *Md*, Cc	*castor*	*Drosophila melanogaster* chr3R:5,713,291-5,733,135 upstream spanning 19,844 bp	*Musca domestica* - 2 uCSBs	Not shown
				*Ceratitis capitata* - 3 uCSBs	
Diptera	*Dm*, *Md*, *Cc*	*Dscam2^c^*	*Drosophila rnelanogaster* chr3L:7,180,410-7,230,562 upstream spanning 50,152 bp	*Musca domestica* - 0 uCSBs	Not shown
				*Ceratitis capitata* - 3 uCSBs	
Diptera	*Dm*, *Md*, *Cc*	*wingless*	*Drosophila melanogaster* chr2L: upstream & intronic spanning 39,780 bp	*Musca domestica* - 15 uCSBs	Not shown
				*Ceratitis capitata* — At least 1 uCSB, multiple genomic rearrangements	
Diptera	*Anopheles gambiae (Ag)*, *Aedes aegypti (Aa)*, *Culex pipiens (Cp)*	*Wnt oncogene analog 4* *& wingless*	*Anopheles gambiae* chr3R:41,999,883-42,001,302 intragenic spanning 1,420 bp	*Aedes aegypti* - 4 uCSBs	Fig 2
				*Culex pipiens* - 4 uCSBs	
Diptera	*Ag*, *Aa*, *Cp*	*glass bottom boat*	*Anopheles gambiae* chrX:15,337,999-15,343,860 upstream & intronic *spanning* 6,861 bp	*Aedes aegypti* - none detected	Not *shown*
				*Culex pipiens* - none detected	
Diptera	*Ag*, *Aa*, *Cp*	*ventral veins lacking*	*Anopheles gambiae* chr2L:23,041,706-23-078,560 upstream & downstream spanning 36,855 bp	*Aedes aegypti* - 3 uCSBs	Not shown
				*Culex pipiens - *3 uCSBs	
Diptera	*Ag*, *Aa*, *Cp*	*goosecoid*	*Anopheles gambiae* chr2R:6,546,821-6,560,426 upstream & downstream spanning 13,605 bp	*Aedes aegypti* - none detected	Not shown
				*Culex pipiens* - none detected	
Diptera	*A*g *Aa*, *Cp*	*castor*	*Anopheles gambiae* chr3R:36,186,488-36,219,684 upstream spanning 33,198 bp	*Aedes aegypti* - 3 uCSBs	Not shown
				*Culex pipiens* - 5 uCSBs	
Diptera	*Ag*, *Aa*, *Cp*	*homothorax*	*Anopheles gambiae* chr2R:17,034,707-17,103,053 upstream and intronic spanning 68,346 bp	*Aedes aegypti* - 4 uCSBs	Not shown
				*Culex pipiens* - 4 uCSBs	
Diptera	*Ag*, *Aa*, *Cp*	*Dscam2^c^*	*Anopheles gambiae* chr2L:5,676,096-5,731,491 55,396 bp upstream & intronic	*Aedes aegypti* - 8 uCSBs	Not shown
				*Culex pipiens* - 3 uCSBs	
Hymenoptera	3 Apoidea (bees) & 4 Formicidae (ants)	*glass bottom boot*	*Apis mellifera* Group1:23,064,324-23,070,923 *Wasmannia auropunctata* LD307392.1 40,378-45,552	Five clusters conserved in both ants and bees; 4 clusters conserved in bees only; 1 cluster conserved in ants only	Fig 3
Hymenoptera	7 Apoidea (bees) & 13 Formicidae (ants)*^d^*	*Dscam2^c^*	*Apis mellifera* GroupUn:45,913,123-45,915,930	*Apis*, *Bombus*, *Habropoda*, 2 CSB clusters *& Megachile* 1 CSB cluster	Fig S4
Hymenoptera	7 Apoidea (bees) & 13 Formicidae (ants)	*goosecoid*	*Apis mellifera* Group6:69,716-71,416	*Apis*, *Bombus*, *Habropoda* and *Megachile* -1 CSB	Fig S5A
Hymenoptera	7 Apoidea (bees) & 13 Formicidae (ants)	*castor*	*Wasmannia auropunctata* LD335973.1 523,260-526,337	Ants and Bees 1 CSB cluster, Ants only, 2 CSBs clusters	Fig S5B
Hymenoptera	7 Apoidea (bees) & 13 Formicidae (ants)	*homothorax*	*Apis mellifera* Group5: 7,111,372-7,117,900 *W. auropunctata* 40,378-45,552	3 CSB clusters conserved in ants & bees, 3,138 clusters conserved in only ants or bees	Fig 56
Hymenoptera	*Apis mellifera* (bee), *Atta cephalotes* (ant)	*wingless &* *Wnt oncogene analog 6*	*Apis mellifera* Group1:17,441,047-17,526,896 Upstream, introgenic and intronic spanning 90,099 bp	*Atta cephalotes* - 10 CSB clusters	Not shown

a Phosphoribosylamidotransferase 2.

b Ultra-conserved Conserved Sequence Blocks.

c Down syndrome cell adhesion molecule 2.

d Species listed in Methods Section.

For detection of conserved sequences in mosquitos, we have adapted *EvoPrinter* algorithms to include 22 species of *Anopheles* plus *Culex pipens* and *Aedes aegypti*. Use of *Anopheles* species allows for the resolution of CSB clusters that resemble those of *Drosophila*. Comparison of *Anopheles* with *Culex* and *Aedes*, separated by ∼150 million years of evolutionary divergence (Krzywinski *et al.* 2006), reveals uCSBs shared among these taxa. Although mosquitoes are considered to be Dipterans, uCSBs were identified conserved between mosquito species but these were generally not found in flies.

In addition, we have developed *EvoPrinter* tools for sequence analysis of seven bee and thirteen ant species. Both ants and bees belong to the Hymenoptera order and have been separated by ∼170 million years (Peters *et al.* 2017). Within the bees, *Megachile* and *Dufourea* are sufficiently removed from *Apis* and *Bombus* (∼100 My) (Peters *et al.* 2017) that only portions of CSBs are shared between species: these can be considered to be uCSBs. uCSBs are found that are shared between ant and bee species (Faircloth *et al.* 2015), and these are positionally conserved with respect to their associated structural genes. Finally, we show that ant specific and bee specific CSB clusters that are not shared between the two taxa are in fact interspersed between shared uCSBs.

## Methods

### Sequence curation and alignment

*Drosophila melanogaster* (*Dm*), *Apis mellifera* (*Am*) and *Anopheles gambiae* (*Ag)*, fly, bee and mosquito genomic sequences respectively, were curated from the UCSC genome browser. BLASTn (Altschul *et al.* 1990) was used to identify non-coding sequences within other species not represented in the UCSC genome browser. Where possible, BLAT (Kent 2002) and BLASTn were used in comparing the order and orientation of ultra-conserved sequences in reference species with dipteran, bee and mosquito test species. BLAT was not available for the *Culex* comparison to *Aedes*, but we found that the ‘align two sequences’ algorithm of BLAST, using the ‘Somewhat similar sequences’ (BLASTn) setting, was comparable to BLAT in sensitivity to sequence homology and was useful for this comparison. Similarly, the pairwise sequence alignment program Needle, which uses the Needleman-Wunsch algorithm (Needleman and Wunsch 1970), aligned shorter regions of near identity that could not be seen using other methods.

### Identification and orientation analysis of non-coding conserved sequence

For comparison of *Drosophila* genomic sequence to *Ceratitis* and *Musca*, we first curated a *D. melanogaster* genomic sequence using the BLAT algorithm, verifying the orientation of the downloaded sequence. We then selected *Ceratitis* and *Musca* from the Refseq Genome Database and submitted the *D. melanogaster* sequence to BLAST using BLASTn. The BLAST answer table was sorted by ‘query start position’ and the orientation of the subject sequence with respect to the orientation of the input genomic was verified. Finally, we analyzed the conserved CSBs with respect to a within *Drosophila EvoPrint* of the input sequence.

For analysis of sequence conservation of mosquito, ant and bee genomes we developed EvoPrint algorithms for each taxonomic group. An *EvoPrint* provides a single uninterrupted view, with near base-pair resolution, of conserved sequences as they appear in a species of interest. Prior papers describe protocols for genome indexing, enhanced BLAT alignments and scoring of *EvoPrint* alignments (Odenwald *et al.* 2005; Yavatkar *et al.* 2008). For discovery of mosquito, ant and bee uCSBs, we first selected the sequence to be analyzed from respectively *Anopheles gambiae* or the *Apis mellifera* genome browser using a coding sequence as an anchor for assuring homologous hits. The curated sequences were submitted to either mosquito or bee *EvoPrinterHD* (evoprinter.ninds.nih.gov), and *EvoPrints* were generated as described previously (Odenwald *et al.*, 2005; Yavatkar *et al.*, 2008). For development of EvoPrintHD, in addition to using the original BLAT procedure (Kent 2002; Odenwald *et al.* 2005), we also generated overlapping 9 and 11 Kmers as described previously by (Yavatkar *et al.* 2008), improving the identification of conserved sequences, and these were used in the EvoPrintHD algorithm. For *EvoPrinting* ant genomic sequences, ant sequence homologous to the *Apis* sequence was curated using BLAST against a single ant species (*Atta*, for example). Care was taken to EvoPrint ant species whose region of interest was intact without major sequence interruptions. Ant and bee *EvoPrints* were examined in side-by-side comparison, using the align two sequence algorithm of BLAST to ensure accuracy.

To compare 24 *Anopheles* (*A*), *Aedes* and *Culex* genomes, sequences were obtained from VectorBase (https://beta.vectorbase.org/vectorbase.beta/app/). The mosquito *EvoPrinter* consists of 20 species as follows; 7 species of the Gambiae subgroup and related species *A. christyi* and *A. epiroticus*; 5 species of the Neocellia and Myzomyia series (including *A. stephensi*, *A. maculates*, *A. calcifacies*, *A. funestus* and *A. minimus*); 2 species of the Neomyzomyia series (*A. darius* and *A. farauti*); 2 species of subgenus *Anopheles* (*A. sinensus* and *A. atroparvus*); Nyssoryhynchus and other American species, (*A. albimanus* and *A. darling*); and two species of the subfamily Culicinae (*Aedes aegypti* and *Culex quinquefaciatus*). Mosquito genomes are documented by Holt *et al.* 2002; Nene *et al.* 2007; Reddy *et al.* 2012, and Neafsey *et al.* 2015.

We have also formatted seven bee species for *EvoPrintHD* analysis, including 6 members of the family Apidae and one member of each of the Megachilidae and Halictidae families (see Table S1). In addition, we have formatted 13 ant (Formicidae) species, a diverse family of social insects, for *EvoPrinter* analysis (see Table S1). Among these are eight species representative of the subfamily Myrmicinae, three representatives of the Formicinae, two of the Ponerinae, and one Dolichoderinae. For consistency, we selected a member of the Myrmicinae as input/reference sequence, and species selection was dependent on the integrity and completeness of the sequence.

### Data availability

*EvoPrinterHD* application for Hymenoptera, *Drosophila*, and mosquitos is available at the following URL: https://evoprinter.ninds.nih.gov/evoprintprogramHD/evphd.html?. Instructions for EvoPrintHD are found at https://evoprinter.ninds.nih.gov/evopoverviewHD.htm. Supplemental material available at figshare: https://doi.org/10.25387/g3.12523505.

## Results and Discussion

### Comparative analysis of dipteran non-coding DNA

**Figure 1:**
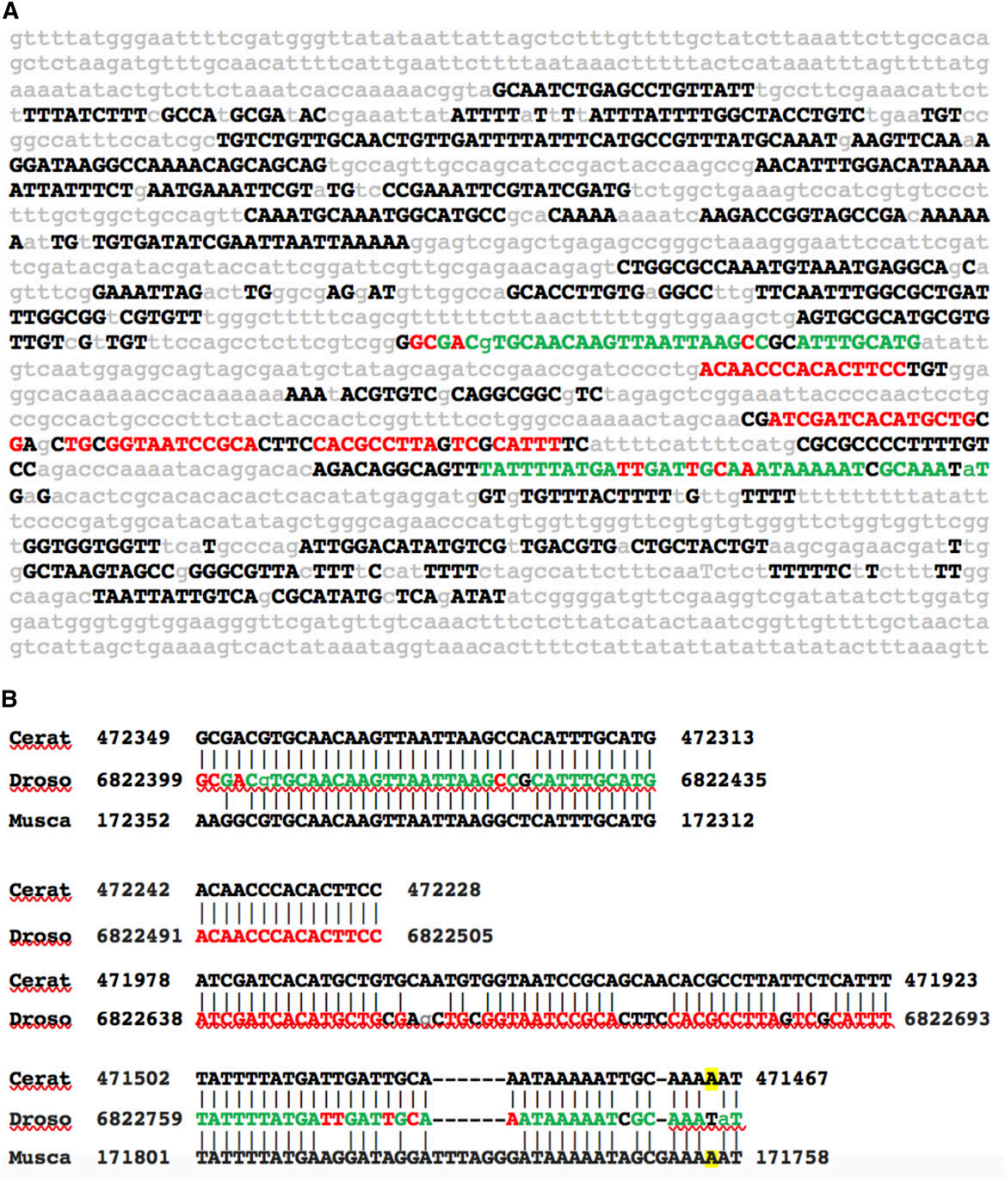
Ultra-conserved sequences shared among a *Drosophila ventral veins lacking* enhancer and orthologous DNA within the *Ceratitis capitata* and *Musca domestica* genomes. A) An *EvoPrint* of the *D. melanogaster *vvl-41 neuroblast enhancer showing 1,775 bp, located 26.6 kb 3′ of the *vvl* transcribed sequence. Capital letters represent bases in the *D. melanogaster* reference sequence that are conserved in *D. simulans*, *D. sechellia*, *D. yakuba*, *D. erecta*, *D. ananassae*, *D. persimilis*, *D. grimshawi*, *D. mojavensis and D. virilis* orthologous DNAs. Lower case gray bases are not conserved in one or more of these species. Conserved sequence blocks (CSBs) shared with *Ceratitis* and *Musca*, as detected using BLASTn, DNA Block Aligner and the *EvoPrinter* CSB aligner are shown in Green text while red bases are shared between *D. melanogaster* and *Ceratitis* but not with *Musca*. B) Two and three-way alignments between of the ultra-conserved CSBs using BLASTn alignments. Green and red font annotations in the *Drosophila* CSBs are as describe above. Yellow highlighted bases in *Ceratitis* and *Musca* are not shared in *Drosophila*. Flanking BLASTn designator numbers indicate genomic sequence positions.

Our previous study of 19 consecutive *in vivo* tested *Drosophila* enhancers, contained within a 28.9 kb intragenic region located between the *vvl* and *Prat2* genes, revealed that each CSB cluster functioned independently as a spatial/temporal *cis*-regulatory enhancer (Kundu *et al.* 2013). Submission of this enhancer field to the RefSeq Genome Database of *Ceratitis capitata* via BLASTn revealed 17 uCSBs; all 17 regions were colinear and located between the *Ceratitis* orthologs of *Drosophila **vvl* and *Prat2* genes. In each case the matches between *Ceratitis* and *Drosophila* corresponded to either a complete or a portion of a CSB identified by the *Drosophila EvoPrinter* as being highly conserved among *Drosophila* species (Kundu *et al.* 2013). Submission of the same *Drosophila* region to *Musca domestica* RefSeq Genome Database using BLASTn revealed 13 uCSBs that were colinearly arrayed within the *Musca* genome. Nine of these *Ceratitis* and *Musca* CSBs were present in both species and corresponded to CSBs contained in several of the enhancers identified in our previous study of the *Drosophila* enhancer field (Kundu *et al.* 2013). The conservation within one of these embryonic neuroblast enhancers, vvl-41, is depicted in Figure 1A and B and Table 1. Each of the CSB elements in vvl-41 that are shared between *Dm* and *Ceratitis* are in the same orientation with respect to the *vvl* structural gene. Figure S1 presents three-way alignments of each of the other eight uCSBs within the *vvl* enhancer field that are shared between *Dm*, *Ceratitis* and *Musca*. The uCSB of vvl-49 in *Ceratitis* is in reverse orientation with respect to the *vvl* structural gene. Many of the uCSBs in Musca are in a different orientation on the contig than in *Dm*, indicating microinversions. One of the two uCSBs in *Ceratitis goosecoid* was in reverse orientation compared to *Drosophila* CSBs, while three of the four uCSBs in *Musca goosecoid* were in reverse orientation (Table 1; data not shown). One uCSB each in *Ceratitis* and *Musca castor* was in reverse orientation compared to *Drosophila castor*. 10 of the 15 uCSBs in the *Musca wingless* non-coding region were in the reverse orientation compared to the orientation in Drosophila, while all uCSBs in *Ceratitis Dscam2* were in forward orientation compared to the orientation in *Drosophila*. We conclude that, except for microinversions, the order and orientation is the same, with respect to flanking genes of highly conserved non-coding sequences in select developmental determinants of *Drosophila*, *Ceratitis* and *Musca*.

Many of the non-coding regions in dipteran genomes contain uCSBs, especially in and around developmental determinants, and many of these are likely to be *cis*-regulatory elements such as those found in the *vvl* enhancer field. Another example is the prevalence of uCSBs found in the non-coding sequences associated the *Dm **hth* gene locus. A previous study identified an ultraconserved region in *hth* shared between *Drosophila* and *Anopheles* (Glazov *et al.* 2005). We have identified additional *hth* uCSBs shared among *Dm*, *Ceratitis* and *Musca*. We examined a 55,100 bp upstream region of *Dm **hth* terminating just after the start of the first exon. We identified a total of 11 CSBs shared between the three species, 5 CSBs shared between *Dm* and *Ceratitis* but not *Musca*, and 6 CSBs shared between *Dm* and *Musca*, but not *Ceratitis* (see Table 1, Figure S2 and data not shown). *Ceratitis* exhibited 4 uCSBs and *Musca* exhibited 8 uCSBs that were in reversed orientation with respect to the *Drosophila* orthologous regions. Additional genes analyzed in this paper were also analyzed for association with uCSBs in *Ceratitis* and *Musca*, and these results are summarized in Table 1. In some cases, for example *wingless* in *Ceratitis*, the presence of uCSBs could not be verified because of the incomplete assembly of the genome, leaving coding sequences and uCSBs on different contigs. In another case, *Dscam2* in Musca, no uCSBs were identified.

*EvoPrint* analysis of *Drosophila **hth* sequences immediately upstream and including the first exon, revealed a conserved sequence cluster (see Figure S2) associated with the transcriptional start site. Fig. S2A illustrates correspondence of the *Dm* conserved region in *Ceratitis* and *Musca*. Two of the longer CSBs were conserved in both *Ceratitis* and *Musca*, one shorter CSB was conserved only in *Musca*, and a second shorter CSB was conserved only in *Ceratitis*. Two and three-way alignments as revealed by BLASTn in a comparison of *Dm*, *Ceratitis* and *Musca* are shown in Figure S2B. Each of the uCSBs was in the same orientation with respect to the *hth* structural gene.

### Discovery of non-coding conserved sequence elements in mosquitoes

*EvoPrinting* combinations of species using A. *gambiae* as a reference species and multiple species from the Neocellia and Myzomyia series and the Neomyzomyia provides a sufficient evolutionary distance from *A. gambiae* to resolve CSBs. Phylogenic analysis has revealed the *Anopheles* species diverged from ∼48 My to ∼30 My (Kamali *et al.*, 2014) while *Aedes* and *Culex* diversified from the *Anopheles* lineage in the Jurassic era (∼145–200 Mya) (Krzywinski *et al.* 2006) or even earlier.

**Figure 2:**
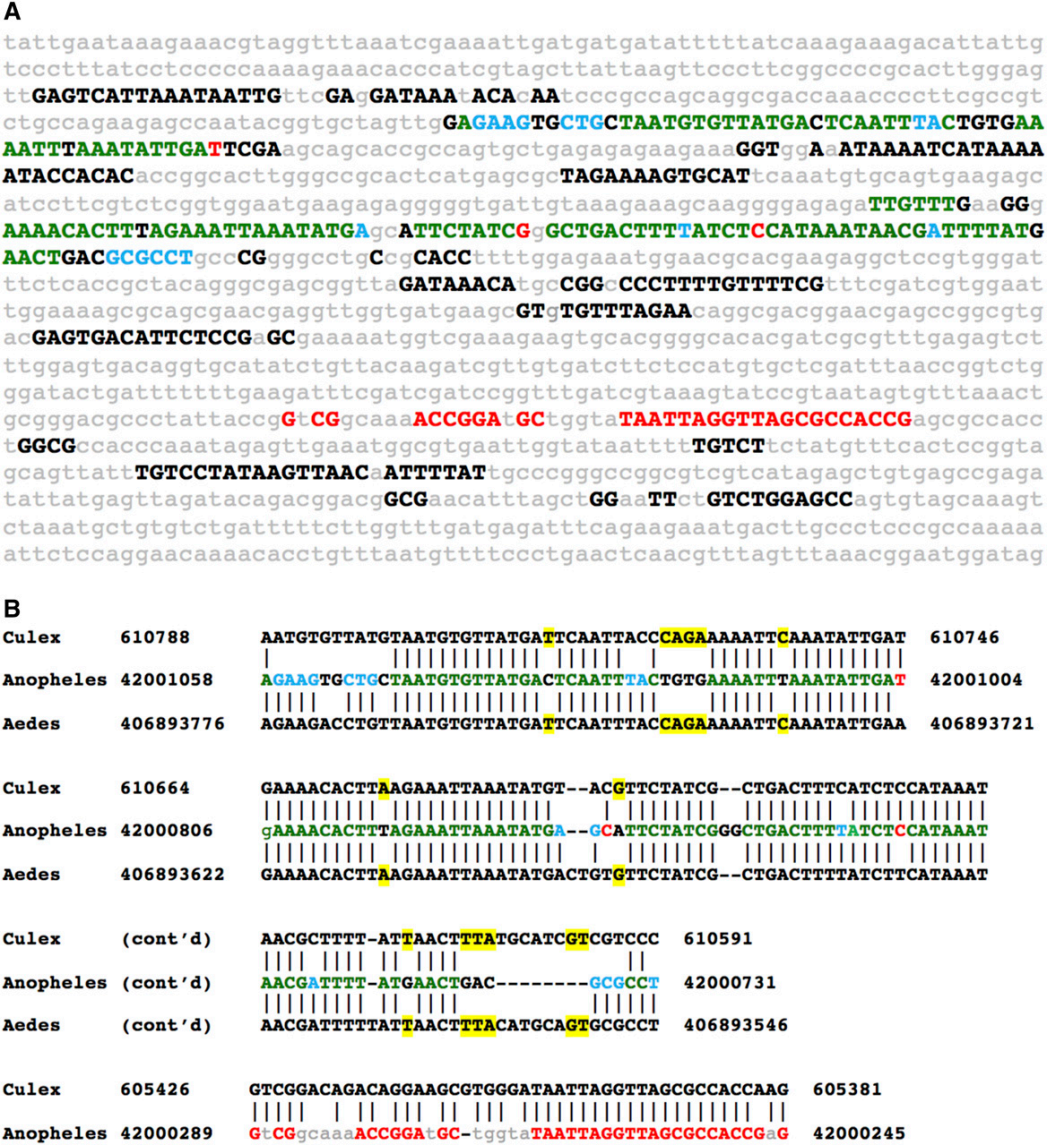
*EvoPrint* analysis of the intragenic region adjacent to the *Anopheles Wnt-4* and *wingless* genes identifies ultra-conserved sequences shared with the evolutionary distant *Culex pipiens* and *Aedes aegypti* genomes. A) *Anopheles gambiae* genomic *EvoPrint* that spans 1,420 bp, located 10.2 kb upstream of the *Wnt-4* gene and 27.5 kb upstream of the *wingless* gene which is transcribed in the opposite orientation of *Wnt-4* transcription. Capital letters (all font colors) represent bases conserved in all or all but one of the following *Anopheles* test species: *A. gambiae-S1*, *A. merus*, *A. melas*, *A. epiroticus*, *A. christyi*, *A. funestus*, *A. culicifacies*, *A. dirus or A. farauti*. Lower case gray letters represent bases that are not conserved in two or more of the *Anopheles* species included in the relaxed *EvoPrint*. Green uppercase bases indicate sequences are conserved in the *Anopheles* species, *Culex pipiens* and *Aedes aegypti*, blue font indicates *Anopheles* sequences that are shared only between *Culex pipiens* but not with *Aedes aegypti* and red font sequences are present only in *Anopheles* and *Culex*. B) To confirm the shared ultra-conserved CSBs, two and three-way BLASTn alignments of the shared sequences are shown. Color coding is as in panel A and yellow highlighted bases in the three-way alignments indicate identity between *Culex* and *Aedes* that is not present in *Anopheles*. Flanking BLASTn designator numbers indicate genome base positions.

We sought to identify uCSBs in selected mosquito developmental genes (Table 1) by comparing *Anopheles* species with *Aedes* and *Culex*. We used non-coding sequences associated with the mosquito homolog of the morphogen *wingless* to discover associated conserved non-coding sequences. Figure 2 illustrates a CSB cluster slightly more than 27,000 bp upstream of the *A. gambiae* *wingless* coding exons. CSB orientation in *A. gambiae* was reversed with respect to the ORF when compared to the orentations of both *Culex* and *Aedes* CSBs. It is noteworthy that this *EvoPrint*, carried out using multiple *Anopheles*, consists of a cluster of CSBs, resembling *EvoPrints* carried out using Drosophila species (Odenwald *et al.* 2005; Brody *et al.* 2007; Kuzin *et al.* 2009; Kuzin *et al.* 2012). This general pattern of CSB clusters separated by poorly conserved ‘spacers’ is prevalent among other developmental determinants in mosquitos (data not show). uCSBs, conserved in *Culex* and *Aedes*, coincide with CSBs revealed by *EvoPrint* analysis of *Anopheles* non-coding sequences. Figure S3 illustrates an *EvoPrinter* scorecard for the non-coding *wingless*-associated CSB cluster described in Figure 2. Scores for the first four species, all members of the gambiae complex, are similar to that of *A. gambiae* against itself, with subsequent scores reflecting increased divergence from *A. gambiae*. *Culex* and *Aedes* are distinguished from the other species by their belonging to a distinctive branch of the mosquito evolutionary tree, the Culicinae subfamily and their low scores against the *A. gambiae* input sequence. No uCSBs were detected associated with *gbb* or *gsc*, while uCSBs were readily detected associated with *vvl*, *cas* and *hth* (Table 1). A single uCSB in *Aedes cas* and two *uCSBs* in *Culex* *cas* exhibited a reverse configuration compared to the uCSBs in *Anopheles* (data not shown). One uCSB in *Culex **vvl* and no uCSBs in *Aedes **vvl* exhibited a reverse configuration compared to the uCSB in *Anopheles* (data not shown). Finally, all uCSBs in *Culex* and *Aedes **hth* were in forward orientation compared to *Anopheles* (data not show). None of the uCSBs shared between *Drosophila*, *Ceratitis* and *Musca* were conserved in mosquitos, with the exception of a single uCSB associated with a 3′UTR (CTTCGTTTTTGCAAGAGGCCCATATAGCTCGCCAA) that is fully conserved in the Dipteran species tested, A possible explanation for this lack of conservation is the observation that mosquitos are only distantly related to Diptera (Wiegmann *et al.* 2011).

### Conserved sequence elements in bees and ants

**Figure 3:**
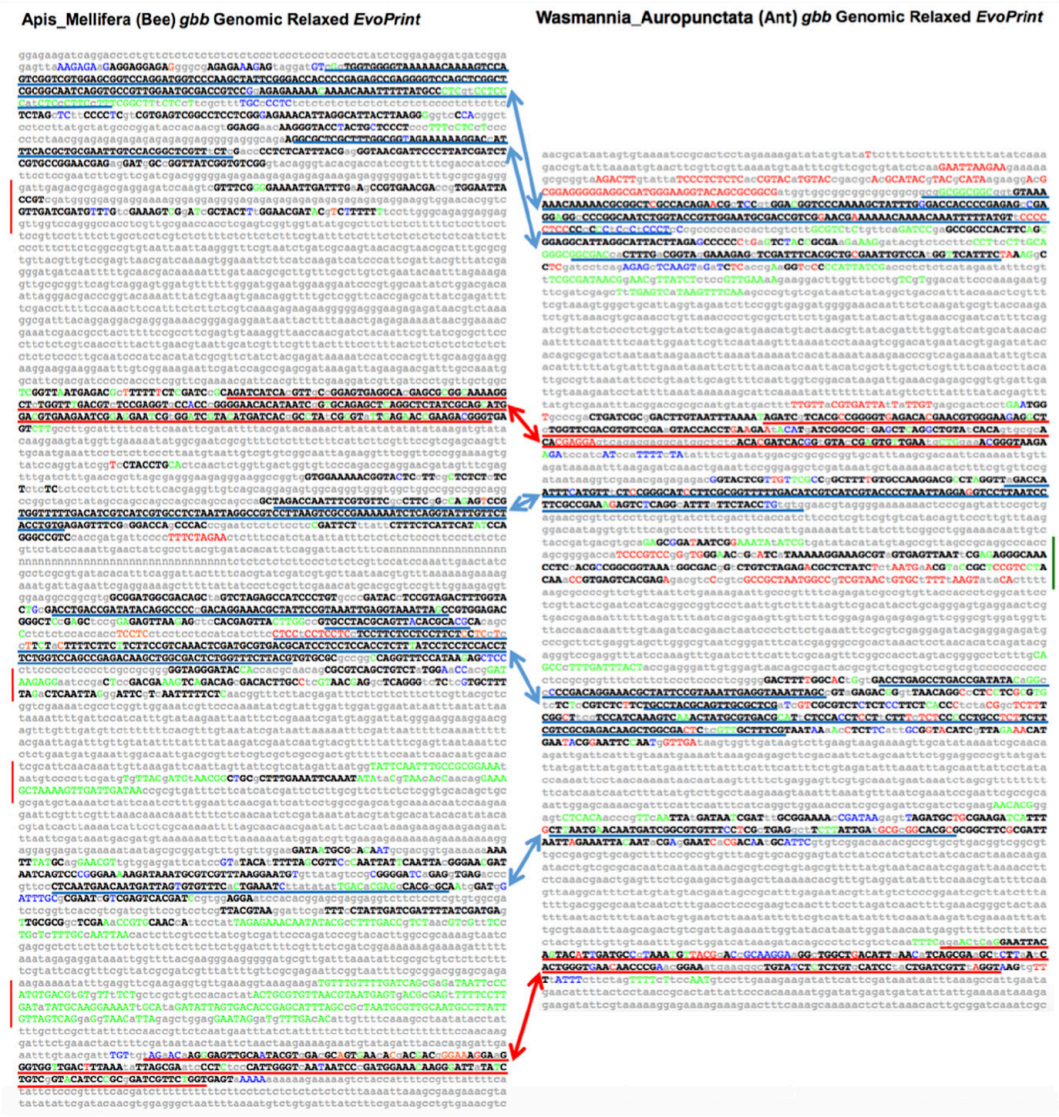
Side-by-side comparison of conserved sequences within the bee and ant *glass bottom boat* loci identify clusters of conserved and species-specific sequences. A) Relaxed *EvoPrint* of *Apis mellifera* genomic DNA that includes the *glass bottom boat* (*gbb*) second and third exons (red underlined sequences) plus flanking intronic sequences (6.6 kb). Black uppercase bases are conserved in all test bee species and colored uppercase bases are conserved in all but one of the color-coded test species: *Bombus terrestrialis*, *Habropoda laboriosa*, *Megachile rotundata* and *Bombus impatiens*. First and second exons sequences underlined red. Blue underlined sequences are homologous to underlined sequences in panel B. Vertical red bars flanking the *EvoPrint* indicate conserved bee-specific sequences that are not found in ants. B) Relaxed *EvoPrint* of *Wasmannia auropunctata* DNA that spans the second and third exons of the *gbb* gene including their flanking intronic sequences (5.1 kb). As in panel A, black uppercase bases are conserved in all test ant species and colored uppercase bases are conserved in all but one of the color-coded species: *Cardiocondyla obscurior*, *Cerapachys biroi* and *Linepithema humile*. Red and blue underlined sequences are respectively homologous coding and non-coding sequences in panel A and the green vertical bar flanking the *EvoPrint* indicates ant-specific conserved sequences that are not found in bees.

Bees and ants are members of the Hymenoptera Order, representing the Apoidea (bee) and Vespoidea (ant) super-families. Current estimates suggest that the two families have evolved separately for over 100 million years (Peters 2017). To identify conserved sequences either shared by bees and ants or unique to each family, we developed *EvoPrinter* alignment tools for seven bee and 13 ant species (see Table S1) and searched for CSBs that flank developmental determinants (Table 1). Three approaches were employed to identify/confirm conserved elements and their positioning within bee and ant orthologous DNAs. First, *EvoPrinter* analysis of bee and ant genes identified conserved sequences in either bees or ants and ultra-conserved sequence elements shared by both families (Figure 3 and Figures S5 and S6). Second, BLASTn alignments of the orthologous DNAs identified/confirmed CSBs that were either bee or ant specific or shared by both (Table 1). Third, side-by-side comparisons of ant and bee *EvoPrints* and BLASTn comparisons revealed similar positioning of orthologous CSBs relative to conserved exons (Figure 3, Figure S7 and data not shown).

To identify conserved sequences within bee species we initially generated *EvoPrints* of the honey bee (*Apis mellifera*) genes using other *Apis* and *Bombus* species. Using *EvoPrints* of the *Dscam2* locus, we resolved clusters of conserved sequences (see Figure S3). *Dscam2* is implicated in axon guidance in *Drosophila* (Millard *et al.* 2007) and in regulation of social immunity behavior in honeybees (Cremer *et al.* 2007; Harpur *et al.* 2019). The *EvoPrint* scorecard (see Figure S4) reveals a high score (close relationship) with the homologous region in the other two *Apis* species. The more distant *Bombus* species score lower by greater than 50%, and *Habropoda* represents a step down from the more closely related *Bombus* species. *Megachile* shows a significantly lower score reflecting its more distant relationship to *Apis mellifera*. The relaxed *EvoPrint* (Yavatkar *et al.* 2008) readout reveals two CSB clusters (see Figure S4). Only one sequence cluster, the lower 3′ cluster, is conserved in all six test species examined, while the 5′ cluster is present in all species except *Megachile*. BLAST searches confirmed that the 3′ cluster was absent from *Megachile*, a more distant species *Dufourea novaeangliae*, and all ant species in the RefSeq genome database (data not shown). BLASTn alignments also revealed conservation of the 3′ cluster in *D. novaeangliae*, the wasp species *Polistes canadensis* and two ant species, *Vollenhavia emeryi* and *Dinoponera quadriceps*.

*EvoPrinter* analysis of bee and ant genes that are orthologs of *Drosophila* neural development genes *goosecoid* (*gsc*) and *castor* (*cas*) revealed conserved non-coding DNA that is unique to either bees or ants or conserved in both (see Figure S5). EvoPrints of the Hymenoptera orthologs identify non-coding conserved sequence clusters that contained core uCSBs shared by both ant and bee superfamilies, and these uCSBs are frequently flanked by family-specific conserved clusters. For example, analysis of the non-coding sequence upstream of the *Wasmannia auropunctata* (ant) *cas* first exon identifies both a conserved sequence cluster that contains ant and bee uCSBs and an ant specific conserved cluster that has no counterpart found in bees (see Figure S5B and data not shown). It is likely that the ant specific cluster was deleted in bees, since BLASTn searches of *Wasmannia* against the European paper wasp *Polistes dominula* reveals conservation of a core sequence corresponding to this cluster (data not shown). The combined evolutionary divergence in the *gsc* and *cas* EvoPrints, accomplished by use of multiple test species, reveals that many of the amino acid codon specificity positions are conserved while wobble positions in their ORFs are not (see Figure S5). The lack of wobble conservation indicates that the combined divergence of the test species used to generate the prints afford near base pair resolution of essential DNA.

Cross-group/side-by-side bee and ant comparison of their conserved DNA was performed using bee specific and ant specific *EvoPrints* and by BLASTn alignments (Figures 3 and Figure S6 and data not shown). Figure 3 highlights the conservation observed among bee and ant exons and flanking sequence of the *glass bottom boat* (*gbb*, *60A*) locus of *Apis melliflera EvoPrinted* with four bee test species (panel A) and the *Wasmannia auropunctata **gbb* locus *EvoPrinted* with three ant species (panel B). Position and orientation of these CSB clusters and uCSBs is conserved. Coding sequences are underlined red, non-coding homologous regions are underlined blue, and novel CSBs present in either ants or bees but not both are indicated by the vertical lines to the side of each *EvoPrint*. Similarly, *EvoPrinting* a single exon and flanking regions of the *Apis mellifera **homothorax* locus with four bee species and generating an ant specific *EvoPrint* of the orthologous ant sequence of the *Ooceraea biroi **homothorax* locus with ten other ant species, reveals CSBs that are conserved in both *Apis* and *Ooceraea*, as well as sequences that are restricted to one of the two Hymenopteran families (see Figure S6).

CSBs associated with *wingless* in *Apis mellifera* and *Atta cephalotes* (Table1) were grouped in clusters similar to those found in *Drosophila* species (Yavatkar *et al.*, 2008 and Kundu *et al.* 2013).

## Summary

This study describes the use of *EvoPrinter* to detect the presence of ultraconserved non-coding sequences in flies, including *Drosophila* species, *Ceratitis* and *Musca*, in mosquitos and in Hymenoptera species. uCSBs of the three fly taxa have, for the most part, maintained their linear order suggesting a functional constraint on the order of regulatory sequences. For mosquitos, an older taxon than that of flies and the Hymenoptera, uCSBs are found to be shared between *Anopheles*, *Culex* and *Aedes*. Importantly, in Hymenoptera, we found uCSBs within clusters of conserved sequences shared between ants and bees. This conservation of core sequences in enhancers suggests that these morphologically divergent taxa share common regulatory networks. Our approaches to detection of uCSBs in flies, mosquitos and ants and bees will lead to a greater understanding of their evolutionary origin and the function of their conserved non-coding sequences. Knowledge of clusters of CSBs and of uCSBs is an important tool for discovery of the core elements of enhancers and their sequence extent.

In most cases both nBLAST and the *EvoPrinter* algorithm had similar sensitivities and gave comparable results. However, we recommend that the two techniques should be used in conjunction with one another to enhance CSB and uCSB detection. For example, by using both approaches, we discovered uCSBs that were identified by one tool but not both. The advantage of *EvoPrinter* is the presentation of an interspecies comparison as a single sequence, while the advantage of nBLAST is that it provides a sensitive detection of sequence homology in a one-on-one alignment. EMBOSSED Needle alignment gives an even more sensitive detection of shorter sequences and is of use once BLAT or *EvoPrinter* has been used to discover shared CSBs and/or CSB clusters.
